# Electric Wire in the Urinary Bladder: Surgical Challenges and Comprehensive Literature Insights

**DOI:** 10.3390/diagnostics14242825

**Published:** 2024-12-15

**Authors:** Daniel Porav-Hodade, Raul Gherasim, Ciprian Todea-Moga, Tibor Reman, Bogdan Ovidiu Feciche, Kosza Hunor, Madalin Guliciuc, Mártha Orsolya Katalin Ilona, Ioan Coman, Nicolae Crisan

**Affiliations:** 1 Department of Urology, George Emil Palade University of Medicine, Pharmacy, Science, and Technology of Târgu Mureș, 540139 Târgu Mureș, Romania; daniel.porav-hodade@umfst.ro (D.P.-H.); raul-dumitru.gherasim@umfst.ro (R.G.); tibor.reman@umfst.ro (T.R.); orsolya.martha@umfst.ro (M.O.K.I.); 2 Department of Urology, Clinical County Hospital Mures, 540136 Târgu Mures, Romania; 3 Department of Urology, Faculty of Medicine and Pharmacy, University of Oradea, 410087 Oradea, Romania; 4 Department of Urology, Emergency County Hospital Oradea, 410169 Oradea, Romania; 5 Clinic of Urology, Miercurea Ciuc County Hospital, 530173 Miercure Ciuc, Romania; kosza@yahoo.com; 6 Department of Surgery, Faculty of Medicine, “Dunarea de Jos” University Galati, 800201 Galati, Romania; guliciuc.madalin@gmail.com; 7 Department of Urology, Iului Hatieganu University of Medicine and Pharmacy, 400012 Cluj-Napoca, Romania; jcoman@yahoo.com (I.C.); nicolae.crisan@umfcluj.ro (N.C.)

**Keywords:** electric cable, knot, bladder, urethra, laparoscopy, polyembolokoilamania

## Abstract

Background/Objectives: An electric wire inserted into the bladder or urethra presents a specific challenge that is frequently encountered in such cases: the potential formation of a tight knot, making extraction nearly impossible. The primary objective of this study was to share our personal experience with patients who had intravesical electric cable insertions and to provide an extensive literature review, offering detailed insights into the various strategies reported for managing such foreign body cases. Methods: Of the four cases with a foreign body in the lower urinary tract, two involved patients aged 19 and 53, respectively, who had inserted an electric cable. During their attempt at self-removal, they developed an intravesical knot, as confirmed by radiographic imaging. Results: In the first case, a bipolar approach was used: a cystoscope was inserted transurethrally into the bladder alongside the cable, while a laparoscopic trocar was introduced suprapubically. Using laparoscopic scissors, the cable was successfully cut and removed. In the second case, due to the cable’s size, a direct cystotomy was performed. At the 3-month follow-up, the uroflowmetry results were normal for both patients. A psychiatric evaluation revealed no abnormalities in the first patient, while the second patient was diagnosed with polyembolokoilamania. Conclusions: The removal of self-inserted electric cables from the urethra and bladder is a challenging procedure, often requiring the urologist’s creativity to prevent potential complications. Many cases can be resolved endoscopically; however, even this minimally invasive approach must be tailored to each case to provide the most suitable solution for the patient.

## 1. Introduction

Self-inserted foreign bodies in the urethra and bladder consistently present considerable challenges in both diagnosis and treatment. The combination of patient reluctance to seek medical care due to embarrassment, diverse clinical presentations, and limitations in imaging poses considerable obstacles. Once diagnosed, selecting the appropriate surgical approach is critical to minimizing the risks and complications. Although the reported cases in the literature are relatively rare, the range of objects involved is remarkably diverse. Besides electric wires, items such as pencils, pens, batteries, nail scissors, and round magnets have all been documented as being inserted into the urethra [1,2,3,4,5,6,7]. The majority of these cases occur predominantly in males, likely due to both a higher frequency of foreign body insertion and the increased difficulty in self-removal owing to anatomical differences [4,8]. 

The electric wire presents a specific challenge that is common to all cases: its relatively long length. Patients typically avoid choosing a short wire due to fear of it becoming stuck in the urethra. Once inserted through the urethra and into the bladder, the wire often bends and forms a knot due to its length. Attempts to remove it subsequently tighten the knot, making extraction impossible.

The age range of the patients involved in such cases is vast, with reports starting from as young as 4 years old [9], where curiosity is often the primary cause, to older adults [10], where psychiatric conditions or unconventional impulses are more commonly implicated. The motivations for inserting foreign objects into the urinary bladder vary widely, encompassing curiosity, sexual habits (such as sexual arousal, masturbation, and exploratory impulses), self-treatment for strictures, and psychiatric disorders, especially in cases involving intoxication [11,12,13,14,15].

Most patients present to the urology emergency department due to their inability to remove the wire, often accompanied by symptoms such as urethralgia, hypogastric pain, dysuria, frequency, urgency, hematuria, or even acute urinary retention [16,17]. In cases where the wire is fully inserted into the bladder, patients may delay seeking medical attention due to embarrassment, eventually presenting only when complications like urinary tract infections or bladder stones develop around the foreign object [9,18]. 

Imaging studies, along with the appearance and material of the electric wire, are crucial factors in determining the treatment approach, which can range from simple manual extraction to endoscopic procedures or, in some cases, open surgery. 

Effectively managing such cases demands a thorough understanding of the underlying causes, accurate diagnostic approaches, and best treatment strategies to prevent complications, especially trauma to the urethra and bladder.

The primary aim of this study was to present our personal experience in diagnosing and managing patients with intravesical electric cable insertions, focusing on the practical approaches and challenges encountered. Furthermore, this article provides an extensive literature review, offering detailed insights into various strategies reported for managing such foreign body insertions.

The study was approved by the Ethics Committee of the Clinical County Hospital Mures, 540136 Târgu Mures, Romania (16673/28.10.2024), and conducted in accordance with the Declaration of Helsinki.

## 2. Materials and Methods

**Figure 1:**
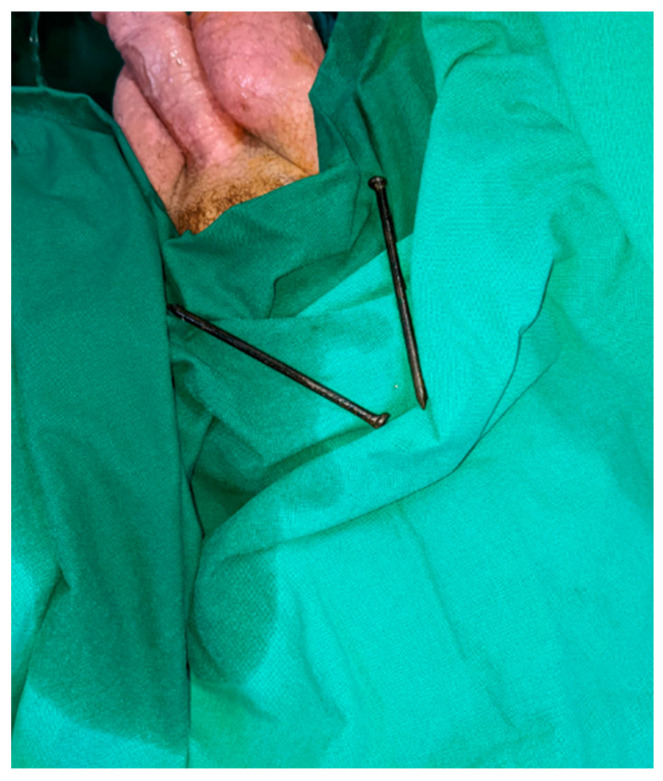
Two nails inserted into the urethra and removed endoscopically using a foreign body clamp.

Between January 2002 and September 2024, we investigated four male patients with foreign bodies introduced into the urethra. These objects included electric wires (two patients), nails (one patient) (Figure 1), and a candle (one patient). Excluding the patients with electric wires, the other two patients underwent successful extraction under local anesthesia in an outpatient setting. For the patients with intravesical electric wires, the surgical approach was different, prompting a detailed discussion on these cases below.

The patients were 19 years old (Case 1) and 53 years old (Case 2). Both presented to the urology department with symptoms of urethral pain, hematuria, and urgency. Additionally, Case 2 exhibited urinary retention (Table 1).

**Figure 2:**
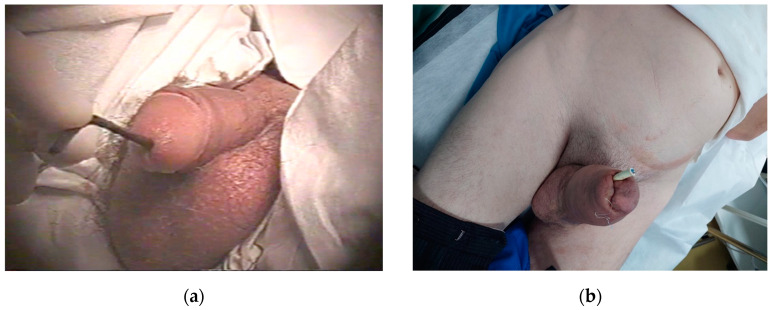
Electric cable inserted into the urethra and visible at the urethral meatus: (**a**) Case 1, the cable had a diameter of approximately 5–6 mm; (**b**) Case 2, the cable diameter was about 10 mm. Additionally, this patient exhibited induration of the penis due to a prior penis kanamycin injection.

On clinical examination, both patients had an electric wire protruding from the urethra. In our two cases, the time from insertion to presentation to the urologist was 1 day for the first patient and approximately 12 h the second patient. This information has been added alongside an appropriate reference. The electric wire in Case 1 had an approximate diameter of 5–6 mm and was reportedly inserted for self-catheterization. The external diameter of the wire in Case 2 was around 10 mm and was inserted for sexual arousal (Figure 2). According to the patients’ histories, neither had psychiatric disorders. Notably, Case 2 had a previous similar episode, where a straw was removed without subsequent psychiatric evaluation. During the local examination of Case 2, edema of the penis was observed. The cause of this edema was self-injection of kanamycin for penile augmentation, performed four months prior, without any ulcerations present in the area.

None of the cases showed signs of an acute abdomen upon clinical evaluation. 

During the digital rectal examination, the knot was palpable near the apex of the prostate.

In both cases, gentle attempts to extract the electric wires were unsuccessful.

Ultrasound examinations for both cases confirmed the presence of an intravesical foreign body. Kidney, ureter, and bladder (KUB) X-rays were also performed for both patients.

To remove the electric cable in both cases, surgical intervention was performed under an epidural anesthesia. The approach was guided by radiological findings and the diameter of the cable visible at the urethra.

## 3. Results

**Figure 3:**
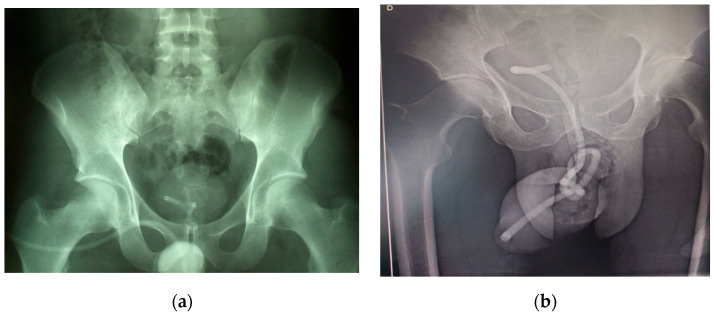
KUB X-ray showing the radiopaque appearance of the intravesical cable: (**a**) in Case 1, the knot is located at the projection area of the bladder neck, (**b**) while in Case 2, the knot is positioned more caudally. This is a figure. Schemes follow another format.

Both cases presented with radiological findings of an intravesical electric cable featuring a knot and a loop (Figure 3). In Case 1, the knot had an approximate diameter of 15 mm, while in Case 2, the diameter was about 20 mm. Ultrasound imaging revealed a knot within the bladder in Case 1, but it was not visible in Case 2. A CT scan was not performed.

Given the position and size of the knots, the therapeutic decision was to proceed with an endoscopic intervention in Case 1 and an open surgical approach in Case 2.

Preoperative prophylactic antibiotic treatment was administered in both cases and continued for 7 days (Table 2).

**Figure 4:**
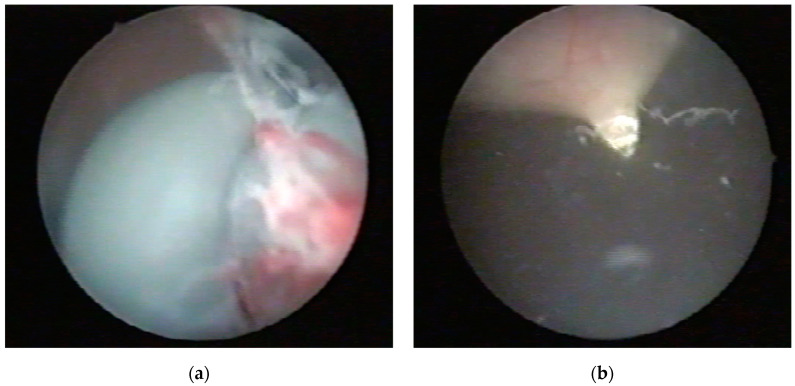
Case 1—cystoscopic view: (**a**) Presence of the knot at the bladder neck; (**b**) Under cystoscopic guidance, a 10 mm laparoscopic trocar was introduced into the bladder suprapubically.

**Figure 5:**
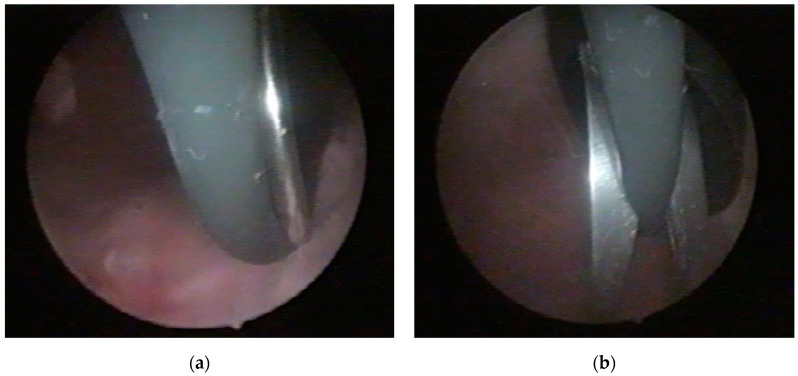
Case 1—cystoscopic view: (**a**,**b**). The loop of the electric cable was cut using scissors inserted through the laparoscopic trocar.

For Case 1, an endoscopic procedure was performed under an epidural anesthesia. A cystoscopy, conducted with a 16 Ch cystoscope inserted alongside the cable into the bladder, did not reveal any lesions or bladder perforations. Inside the bladder, the electric cable was observed with a knot positioned at the bladder neck and a large loop (Figure 4a). Under cystoscopic guidance, a 10 mm laparoscopic trocar was percutaneously inserted into the bladder at the suprapubic level (Figure 4b). A pair of scissors was introduced through the trocar, allowing for the sectioning of the cable at both the loop and the knot (Figure 5). The fragments were then extracted under endoscopic guidance through the laparoscopic trocar. The procedure was completed with the insertion of an 18 Fr bladder catheter following the removal of the trocar.

For Case 2, the cable was removed via a classic cystotomy. We performed a suprapubic open cystotomy through a 5 cm skin incision under an epidural anesthesia, allowing for the complete extraction of the electric cable and knot from the bladder, despite the knot being located at the level of the prostate. 

In both cases, the postoperative recovery was uneventful. At the 3-month follow-up, the patients’ urine cultures were sterile, there was no evidence of urethral stricture, and the uroflowmetry values were within normal ranges (Table 3).

At the 3-month follow-up, Case 2 showed no penile lesions related to the prior kanamycin injection. The patient was advised to undergo a urological evaluation every 6 months or sooner if any lesions developed on the penis.

The psychiatric evaluation for Case 1 was normal, while for Case 2, the assessment led to a diagnosis of polyembolokoilamania (the act of inserting foreign bodies into orifices).

## 4. Discussion

We conducted a search in the following electronic databases: Web of Science, Scopus, and PubMed, using the following search terms identified in the title or abstract: electric wire, electric cable, USB cable, USB wire, telephone wire, and telephone cable. Each term was combined with either bladder or urethra, resulting in 12 search pairs (e.g., electric wire and bladder). The search included all the studies published between 1975 and 2024.

**Figure 6:**
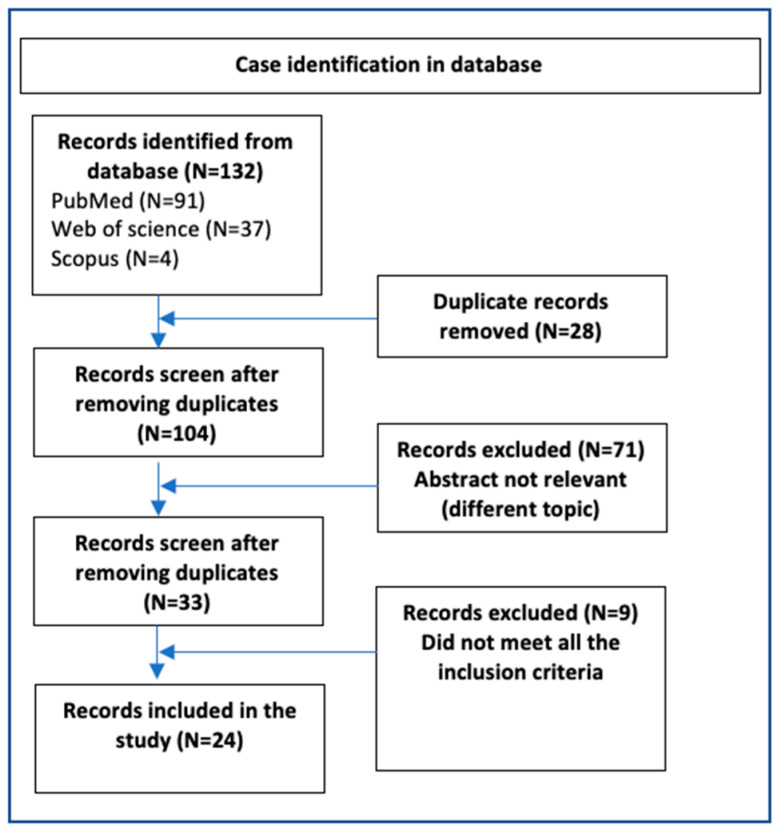
Flow chart showing the detailed procedure for the inclusion or exclusion of studies.

*Inclusion Criteria.* The inclusion criteria specified that articles must focus on male patients and provide detailed information on the following aspects: patient age, type and position of the cable, reason for insertion, imaging findings, surgical procedures performed, presence of a psychiatric evaluation, and the psychiatric diagnosis, if applicable. Studies lacking any of these parameters were excluded from the analysis (Figure 6). The thickness of the cables was not assessed in any of the included studies; therefore, this factor was not incorporated as an inclusion criterion in the database search.

The 24 articles reviewed presented a total of 28 cases. The primary motivation for cable insertion was sexual in nature. Except for five cases where the cable was confined to the urethra, in all other instances, the cable was also present in the bladder. Imaging evaluation was performed using CT in only three cases, while for all others, a KUB X-ray was utilized. Detailed data regarding these cases are presented in Table 4.

In the two cases presented in this article, patients sought emergency care due to symptoms (with acute urinary retention in one case) and the visible presence of the cable at the urethral meatus. Typically, in such situations—especially when the cable is fully inserted into the urethra or bladder—patients often delay seeking medical assistance due to feelings of guilt and humiliation. As a result, they tend to present late, often with complications such as calcification, urethral fistula, recurrent urinary tract infections, or even sepsis. This delay makes the treatment of self-inserted foreign bodies in the urethra significantly more challenging.

In both cases, the electric wires contained metallic components, making them radiologically visible. Considering that the patient showed no signs of an acute abdomen or the migration of the foreign body to other organs, we did not deem it necessary to perform a CT scan. For confirming the position and shape of the electric cable and any associated knot, an ultrasound evaluation alone is insufficient. Confirmation can be achieved using a KUB X-ray, as these cables are radiopaque. Computed tomography is rarely required but becomes essential if there is suspicion that a urethral foreign body has migrated to the adjacent organs or the peritoneum [36].

Antibiotic treatment is mandatory in these cases. Palmer et al. [37] recommend initiating empirical therapy for gram-negative organisms (such as fluoroquinolone or trimethoprim–sulfamethoxazole) prior to the procedure and continuing it for 1 week post-procedure.

To determine the surgical strategy, it is essential to assess both the size and shape of the knot. If the knot is too large, any attempt to remove the wire by traction through the urethra may result in its impaction within the bulbar urethra, making both anterograde and retrograde extraction impossible. In such cases, the only surgical solution is an open urethrotomy. The diameter of the knot can be evaluated using imaging techniques such as X-ray and CT. According to Ashley CW in The Ashley Book of Knots [38], the basic formula for estimating the minimum diameter of a simple knot is approximately twice the diameter of the cable, assuming that the knot is simple and does not involve tight bending or multiple loops. In the case of a more complex knot, the size can increase significantly, often exceeding twice the cable’s diameter, and potentially reaching 3–4 times the original diameter, depending on the knot’s type and complexity.

The treatment approach for the cables inserted into the bladder varies. The primary objective is to prevent urethral trauma, which can range from minor mucosal injury to complete rupture. In most cases, such urethral injuries will result in recurrent urethral strictures.

In rare cases, certain foreign bodies may be expelled spontaneously from the bladder during urination. 

If these cables are knot-free, they can typically be removed from the bladder through gentle traction. This maneuver must be performed carefully, as pulling on long cables increases the risk of the knots’ formation. For knot-free cables that are not externally visible at the urethral meatus, extraction can be achieved using grasping forceps under cystoscopic control. 

The presence of knots within the bladder or urethra significantly alters the treatment approach. In addition to the importance of knot location and diameter, we must also consider the physiological maximum stretch of the external urethral meatus.

The first to describe the diameter of the urethral meatus was Thomson R [39], who indicated that the maximum dilation of the urethral opening is 29.6 Fr, or approximately 0.99 cm. Other authors also noted a maximum urethral meatus opening near this value [40,41]. Therefore, attempting to extract a cable with a diameter exceeding 5 mm (with a corresponding knot size of at least 10 mm) poses a high risk of urethral trauma.

Crawford et al. [38] introduced an extraction technique using a 6 Fr pediatric silicone Foley catheter. Under ultrasound guidance, this catheter is inserted into the urethra alongside the foreign body. Once positioned, 2 mL of sterile saline are introduced into the catheter balloon. Then, under ultrasound visualization, the catheter with the inflated balloon is slowly withdrawn together with the foreign body. This technique is suitable for cases where the knot diameter less than 10 mm.

Another method for removing intravesical foreign bodies, which can also be applied to electric wires, is the Endoloop technique [42]. This method, described by Al-Zubaidi et al. [43], involves endoscopic removal of the foreign body using an Endoloop. A 22 Fr rigid cystoscope is used, and a JJ stent pusher, containing a wire loop at the distal end, is introduced through the working channel of the cystoscope into the urethra. The loop is then maneuvered over the rounded end of the foreign body, tightened around it, and retracted, allowing the cystoscope and foreign body to be withdrawn together.

This technique is particularly suitable for cases where the knot is smaller than 10 mm or when endoscopic grasping forceps cannot be used.

Currently, no instruments can be inserted through the working channel of a cystoscope with the capability to cut a foreign body as hard and thick as an electric cable, except for the use of surgical lasers.

Bedke et al. [44] tested the Holmium:YAG laser for fragmenting both medical objects (catheters, stents) and non-medical materials (wood, steel, graphite). The fragmentation of these objects was successfully achieved in vitro using the Holmium:YAG laser. However, copper wires could not be fragmented with this type of laser, even when powers of 18 to 30 W were applied. Contrary to these findings, in a case described by Agarwal et al. [19], the Holmium laser was successfully used to remove an intravesical telephone cable; however, the electric cable had a very small diameter, as did the copper core.

Although studies have been conducted on the resistance of various guidewires to thulium:YAG laser injury [45], there are currently no studies on the effectiveness of the thulium:YAG laser for fragmenting copper wires.

When the transurethral extraction of these electric cables fails, the only alternative approach is through a suprapubic one. 

In addition to the technique described in this article using a 10 mm laparoscopic trocar, other methods for foreign body removal have been documented, including extraction through pneumovesicoscopy [22,46,47].

Pneumovesicoscopy, also referred to as pneumovesicum, is a minimally invasive urological technique that enables the insertion of a 10 mm (or 5 mm) optical port into a saline-distended urinary bladder under cystoscopic guidance for visualization. After removing the cystoscope and draining the saline from the bladder, CO_2_ is insufflated into the bladder at a pressure of 8–10 cm H_2_O to create a working space. Subsequently, two lateral 5 mm operative ports are introduced under video guidance, allowing instruments to be inserted for attempting to untie or cut the knot.

This technique is generally used in pediatric patients with 5 mm trocars as an alternative to open surgical treatment. Although it shows potential as a viable option for retrieving foreign bodies, the number of trocars introduced inside the bladder and the risk of air embolism [48] have limited its frequent use in the urological armamentarium.

Although the minimally invasive techniques described above provide a viable alternative for this type of foreign body introduced into the urinary bladder, open surgery is sometimes the only effective solution to this problem. For large-knot electric cables located in the urinary bladder, cystotomy is the only viable solution. If the knot is impacted in the urethra, especially in the posterior section, or if it is accompanied by a significant inflammatory response, urethrotomy becomes the only alternative. Although open surgical removal is efficient and quick, it is more invasive, has a higher complication rate, and requires a longer postoperative recovery period [29,49,50]. 

Cystotomy is technically less demanding and has a lower complication rate compared with urethrotomy, which involves a midline perineal or penile approach with a longitudinal incision over the affected urethral segment to expose the foreign body, followed by careful dissection to remove the cable without additional trauma, meticulous urethral repair using fine absorbable sutures, and the placement of a bladder catheter [29]. However, urethrotomy poses significant technical challenges, including precisely locating the cable, achieving adequate exposure while minimizing trauma—especially in cases where the cable is deeply embedded or coiled—and preserving the integrity of the urethral wall by avoiding excessive tissue removal. Potential complications include the development of strictures and infections, arising from the procedure’s invasive nature and the presence of a foreign body.

The motivation for the self-insertion of foreign bodies is extremely varied. In addition to the reasons noted in our cases—such as self-catheterization to relieve urinary symptoms and autoerotic or sexual gratification—many other causes have been documented, including drug intoxication, mental confusion, sexual curiosity, and more.

Angulo-Lozano et al. [51], in a study of 10 cases involving men diagnosed with erectile dysfunction (ED) and the insertion of foreign bodies into the lower urinary tract, concluded that it is essential to conduct a psychiatric evaluation to diagnose or address any underlying psychiatric conditions that may contribute to this behavior, but there is no consensus regarding the necessity of a psychiatric assessment for patients who have self-inserted foreign objects into the urethra [50,52].

Wise [53] describes urethral manipulation as a paraphilia combining elements of sadomasochism and fetishism, wherein the individual can only achieve an orgasm in the presence of this specific fetish. Kenney [52] suggests that the initial episode, which often triggers future occurrences, typically involves the accidental discovery of pleasurable urethral stimulation, which is then replicated for sexual gratification.

Sinopidis et al. [11] introduced the term “internet-induced paraphilia”, describing a new category of sexual behavior in children and adolescents, driven by their access to information through specific websites or direct interactions with other users.

The most well-known group of disorders observed in these patients is polyembolokoilamania [32,50,54]. As previously mentioned, this condition is characterized by the repetitive self-insertion of objects into body orifices. This obsessive–compulsive disorder is often associated with Smith–Magenis syndrome, which includes developmental delays, sleep and behavioral disorders, mild to moderate intellectual disability, delayed speech and language skills, cognitive impairment, and distinctive physical features such as a broad, square-shaped face, deep-set eyes, full cheeks, and a prominent lower jaw [55].

## 5. Conclusions

Although our experience is based on only two cases, which does not allow us to draw definitive conclusions, the cases reported in the literature regarding this topic emphasize that the removal of self-inserted electric cables from the urethra and bladder is a complex procedure, often requiring the urologist’s skill and ingenuity to minimize potential complications. While many cases can be resolved endoscopically, even this minimally invasive approach must be customized to offer the best possible outcomes for the patient. Psychiatric evaluation is recommended for all such patients and becomes mandatory in cases where these self-insertion behaviors are recurrent.

## Figures and Tables

**Table 1:** Characteristics of the evaluated cases.

	Age	Sex	Cable Type	Location	Symptoms	Introduction Reason	Imaging
Case 1	19	male	Electric wire	urethra/ bladder	urethral pain, hematuria, urgency	self-catheterization	KUB X-ray
Case 2	53	male	Electric wire	urethra/ bladder	urethral pain, hematuria, urgency, urinary retention	sexual arousal	KUB X-ray

**Table 2:** Types of surgical approach of the studied cases.

	Bladder Knot	Approach	Type of Surgery	Operating Time	Anesthesia	Antibiotics
Case 1	Yes	Endoscopic	Bipolar endoscopic (urethra) and laparoscopic (suprapubic) Approach	40 min	epidural	Yes, Amoxicillin
Case 2	Yes	Open	Cystotomy	45	epidural	Yes, Ceftazidime

**Table 3:** Postoperative course of the studied cases.

	Approach	Postoperative Bladder Catheterization	Postoperative Psychiatric Evaluation	Hospitalization	3-Month Uroflowmetry
Case 1	Endoscopic	7 days	Yes, psychiatric normal	1 day	Maximum flow rate 19.8 mL/s
Case 2	Open	10 days	Yes, polyembolokoilamania	2 days	Maximum flow rate 17.04 mL/s

**Table 4:** Clinical and surgical details of the studied cases.

	Reference	Age	Cable Type	Cable Position	Introduction Motivation	Imaging	Surgery	Psychiatric Follow-Up
1	Agarwal [19]	18	Electric cable	Bladder, urethra	Sexual gratification	KUB X-ray	Endoscopic holmium	No psychiatric history Foreign body insertion in the past
2	Romero Pérez [17]	14	Video game cable	Bladder, urethra	Curiosity	KUB X-ray	Cystotomy	Psychiatric normal
3	Ahn [20]	30	Electric wire	Bladder, urethra	Masturbation	KUB X-ray	Cystotomy	Psychiatric normal
4	Tuncer [21]	37	Electric cable	Bladder, urethra	Sexual arousal	CT	Endoscopic extraction	Psychiatric normal
5	Garg [16]	21	Electric cable	Bladder, urethra	Sexual arousal	KUB X-ray	Suprapubic nephoscopy	Antipsychotic treatment
6	Ko [22]	30	Electric cable	Bladder, urethra	Sexual arousal	KUB X-ray	Bipolar pneumovesicum	Psychiatric normal
7	Ratkal [13]	30	Electric cable	Bladder, urethra	Self-treatment for stricture	KUB X-ray	Suprapubic extraction	Antidepressants
8	Pal [23]	15	Electric cable	Bladder, urethra	Sexual arousal	KUB X-ray	Cystotomy	Psychiatric normal
9	Pal [24]	13	Mobile charger	Bladder, urethra	Electric plug for sexual pleasure	KUB X-ray	Cystotomy	Polyembolokoilamania
10	Ejstrud [25]	66	Electric cable	Bladder, urethra	Sexual pleasure	KUB X-ray	Laparoscopic, suprapubic, untied knot two clamps	No psychiatric evaluation
11	Ahmed [26]	63	Electric cable	Urethra	Self-treatment	KUB X-ray	Cystoscopic extraction	No psychiatric disorder
12	Soomro [27]	19	Electric cable	Bladder, urethra	Self-insertion	KUB X-ray	Cystotomy	No psychiatric evaluation
		40	Electric cable	Bladder, urethra	Self-insertion	KUB X-ray	Cystoscopy	No psychiatric evaluation
13	Kesieme [28]	35	Electric cable	Bladder, urethra	Sexual arousal	KUB X-ray	Cystotomy	No psychiatric evaluation
14	Mahadevappa [12]	24	Soldering wire	Bladder, urethra	Sexual gratification	KUB X-ray	Cystotomy	Manic-depressive
		38	Electric cable	Bladder, urethra	Masturbation	KUB X-ray	Cystotomy	Psychiatric counselling
		18	Electric cable	Bladder, Urethra	Masturbation	KUB X-ray	Cystotomy	Psychiatric counselling
		48	Telephone wire	Bladder, urethra	Sexual gratification	KUB X-ray	Cystotomy	Psychiatric counselling
15	Song [29]	48	Cell phone charging	Urethra	Sexual arousal, 2 years ago	CT	Open urethrotomy	No psychiatric evaluation
16	Bote [30]	64	Electric cable	Urethra	Self-catheterization	KUB X-ray	Endoscopic extraction	Behavioral disorders, history of introduction of foreign body into the rectum
17	Sinopidis [11]	12	Electric cable	Urethra	Curiosity	KUB X-ray	Gentle traction through the meatus	Psychiatric consultancy Not any mental disorder
18	Amiroune [31]	36	Electric cable	Bladder, urethra	Self-stimulate	KUB X-ray	Endoscopic with urethrotomy (stenosis)	No psychiatric history History of self-inflicted penile strangulation in childhood
19	Guitynavard [14]	19	Telephone cable	Bladder, urethra	Sexual pleasure	KUB X-ray	Endoscopic extraction	Psychiatric counselling
20	Saputra [32]	34	Earphone wire	Bladder, urethra	Masturbating	KUB X-ray	Cystoscopy–grasping forceps	Polyembolokoilamania
21	Loufopoulos [33]	15	USB cable	Urethra	Sexual experimentation	KUB X-ray	Peno-scrotal urethrostomy	No mental disorders
22	Ndiaye [34]	32	Electric cable	Urethra	Genitals manipulations by the partner	KUB X-ray	Approach to the urethra through a perineal incision	History of psychiatric disorders
23	Jamil [35]	18	Electric cable	Bladder, urethra	Sexual gratification	CT	Cystotomy (large bladder stone on the electric cable)	Psychiatric evaluation—not suffering from sexual addiction or autoeroticism
24	Trehan [36]	50	Telephone wire	Bladder, urethra	Sexual gratification	KUB X-ray	Manual extraction	Advice for psychiatric referral

## Data Availability

The data presented in this study are available on request from the corresponding author.
